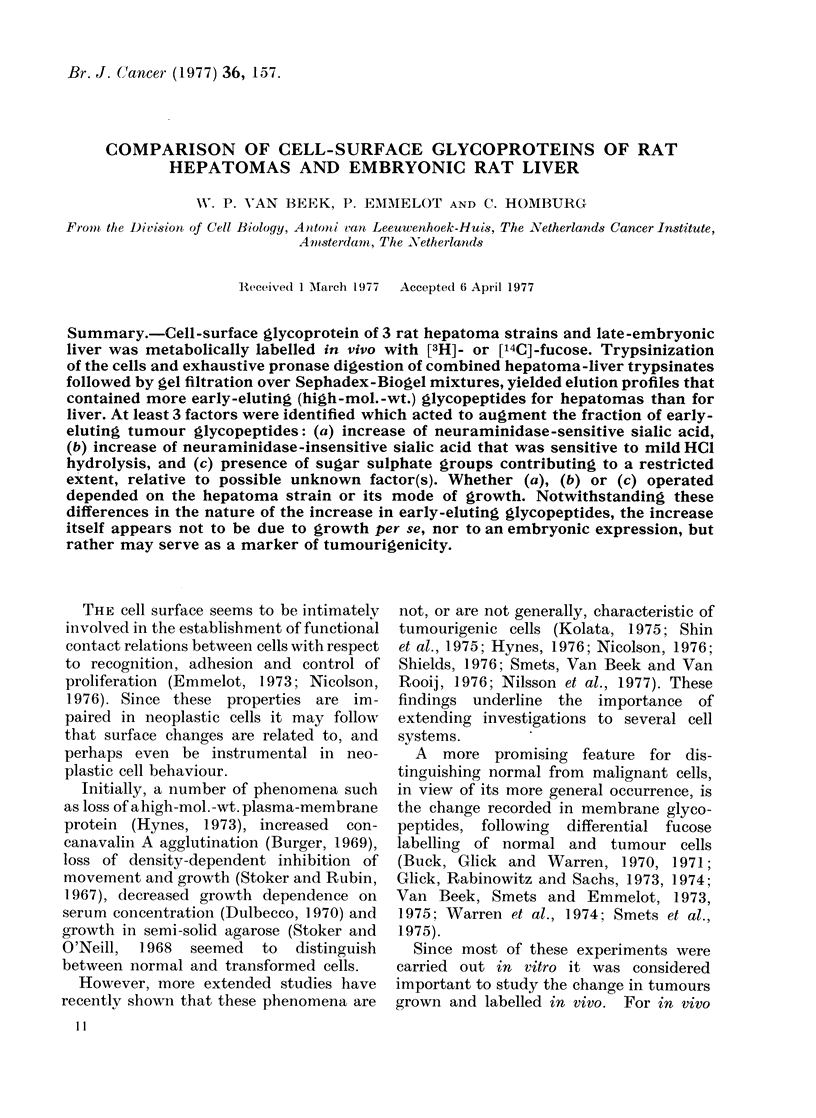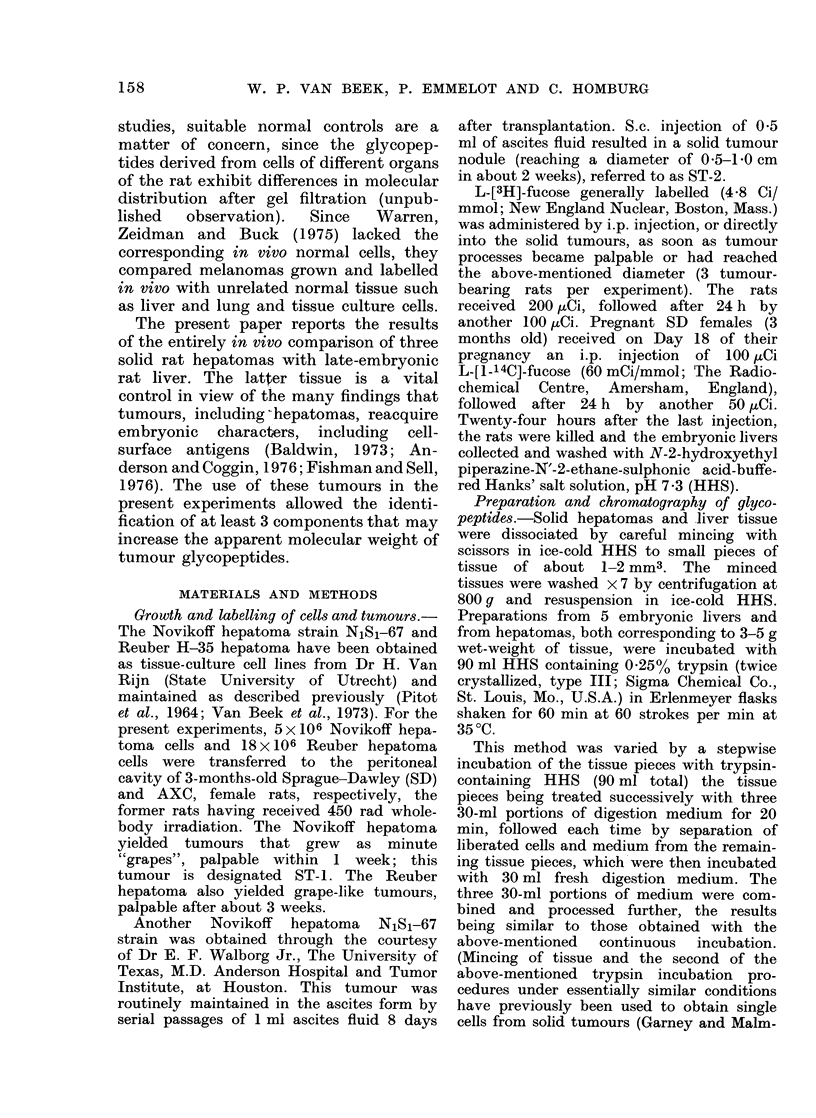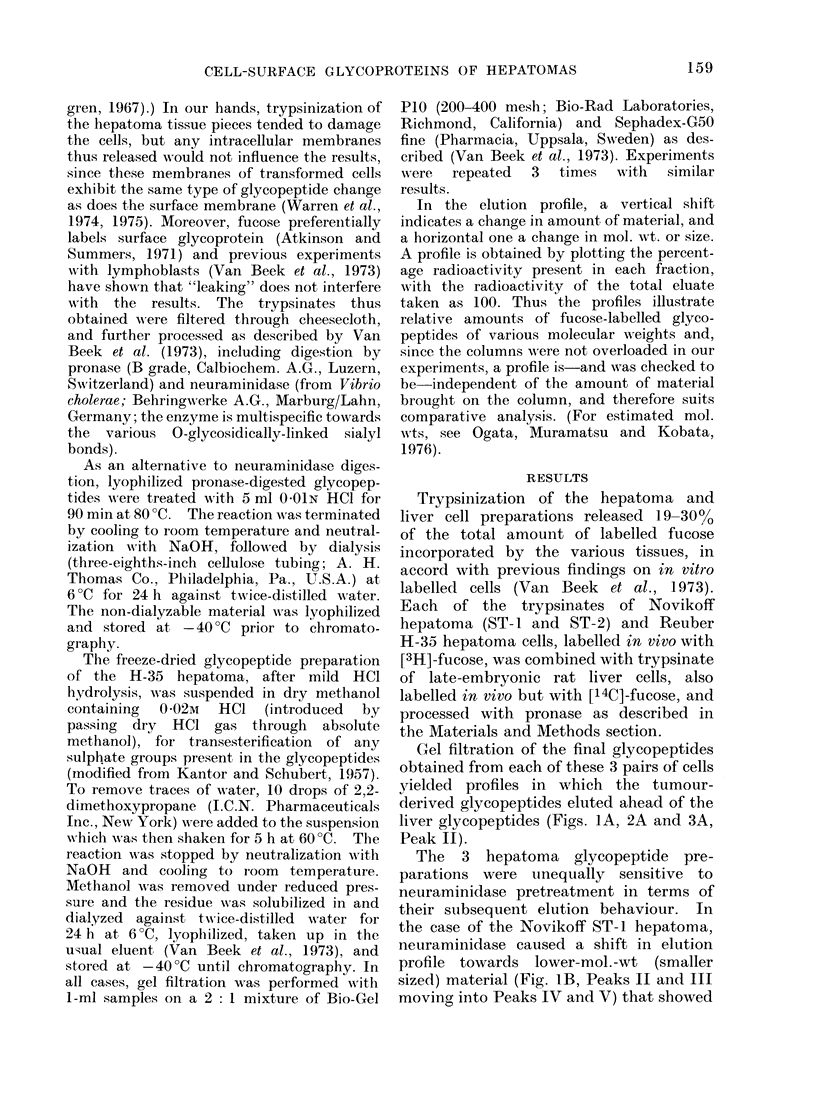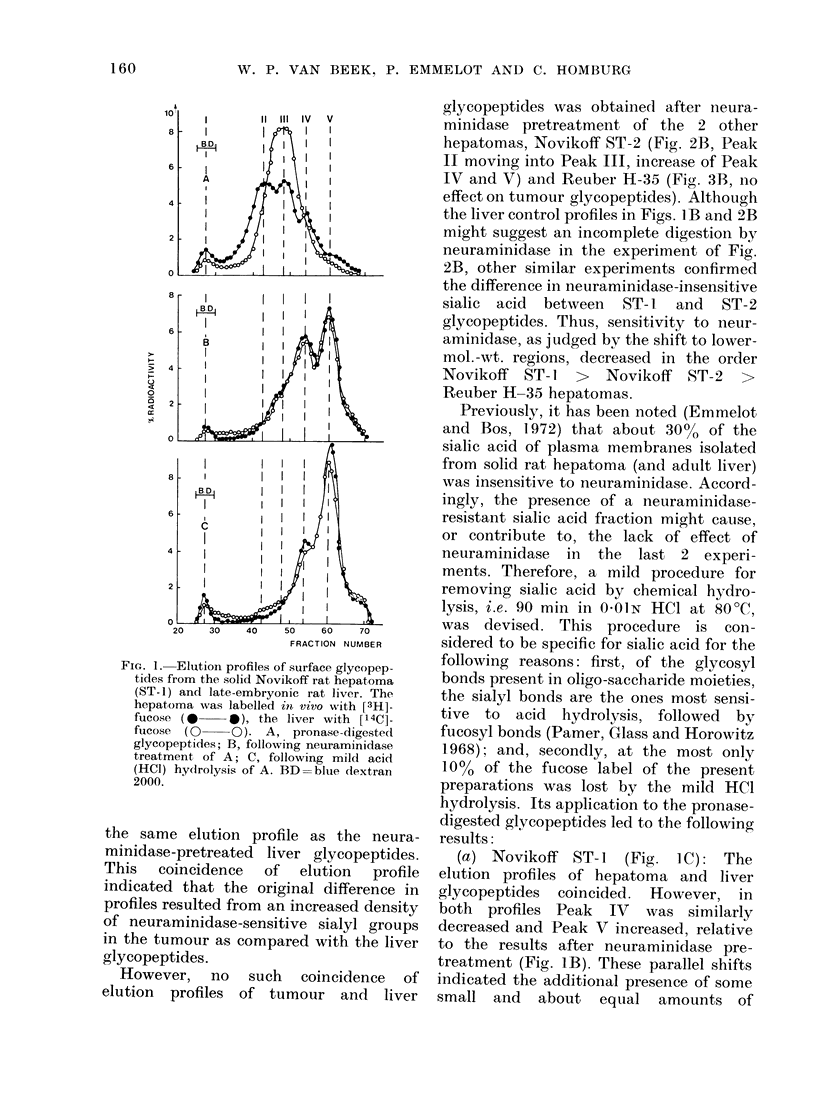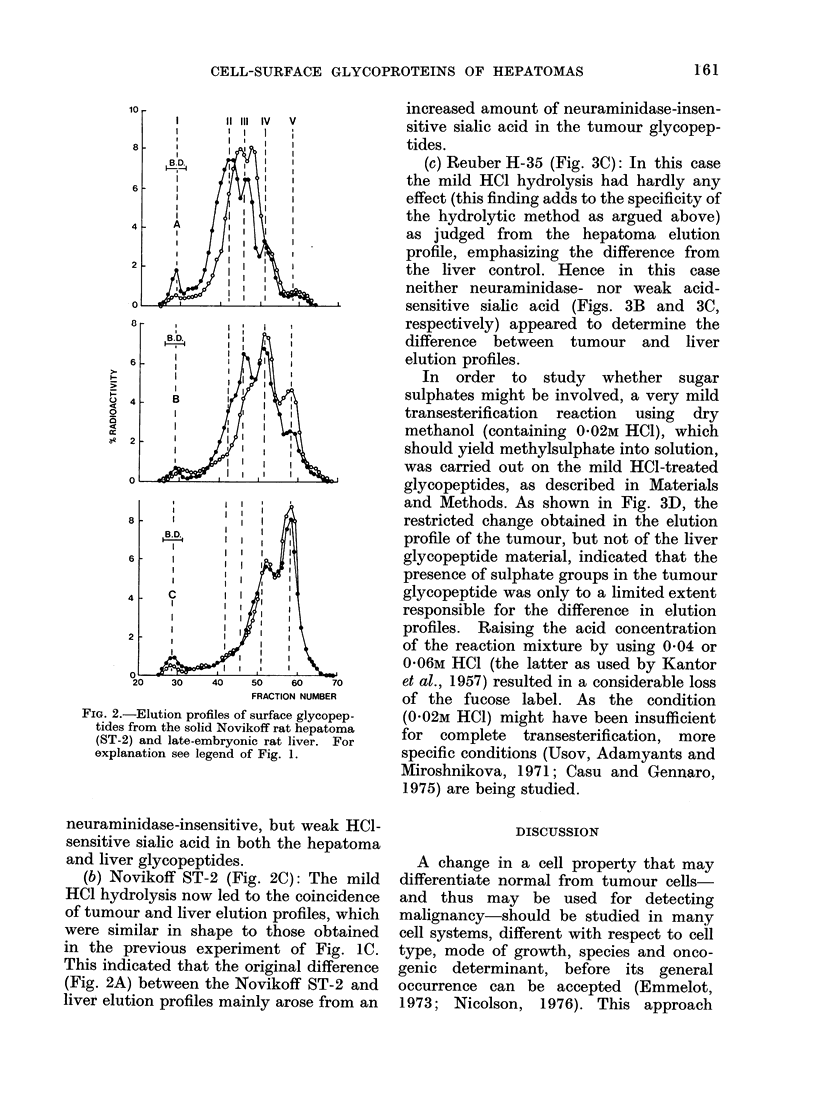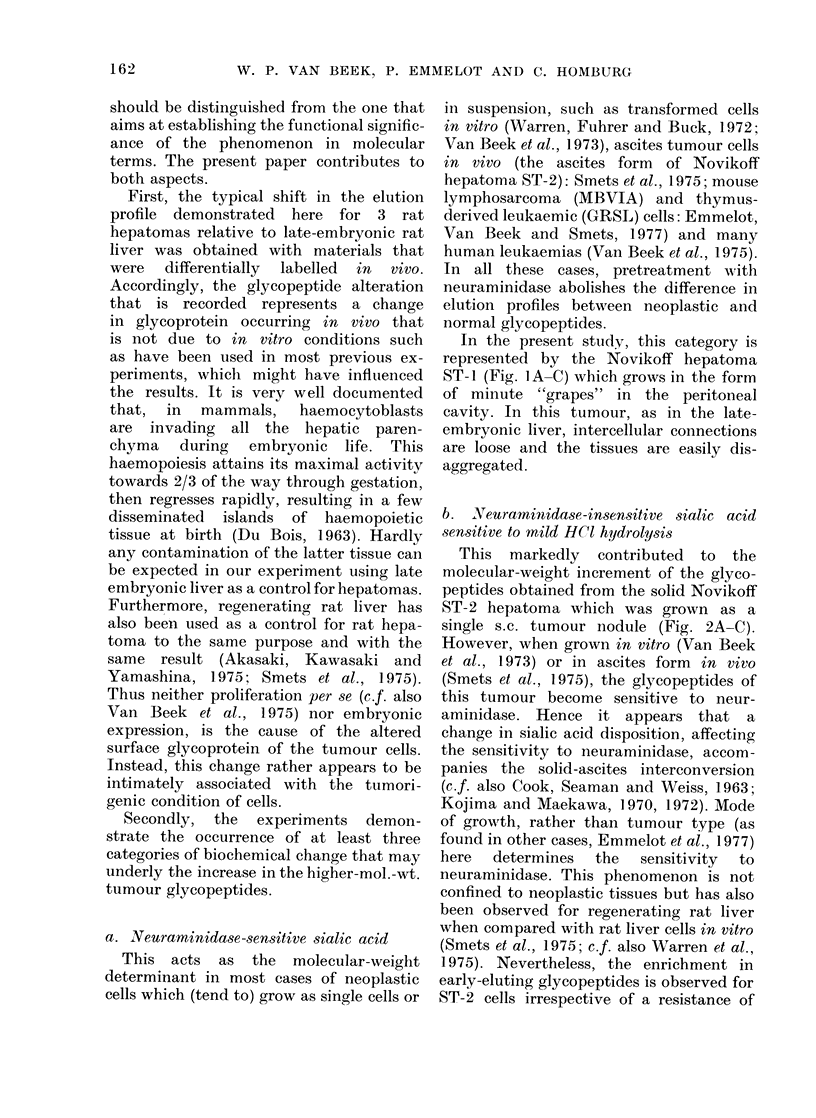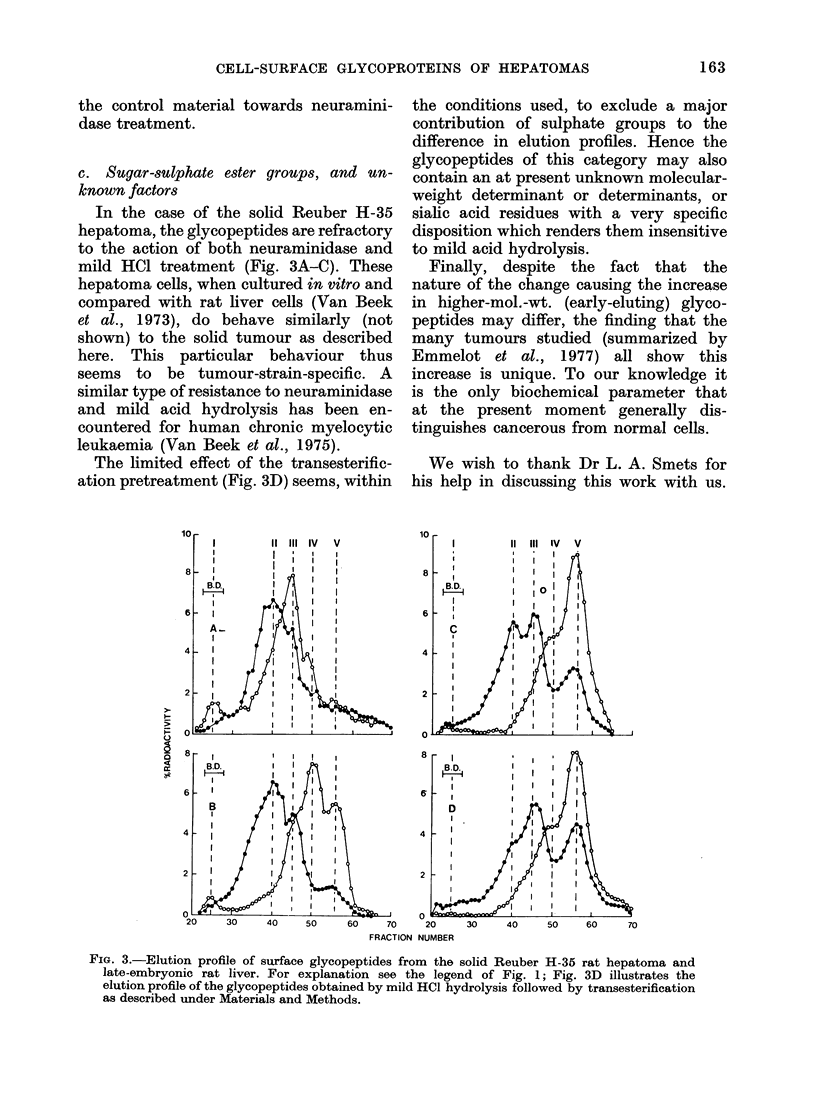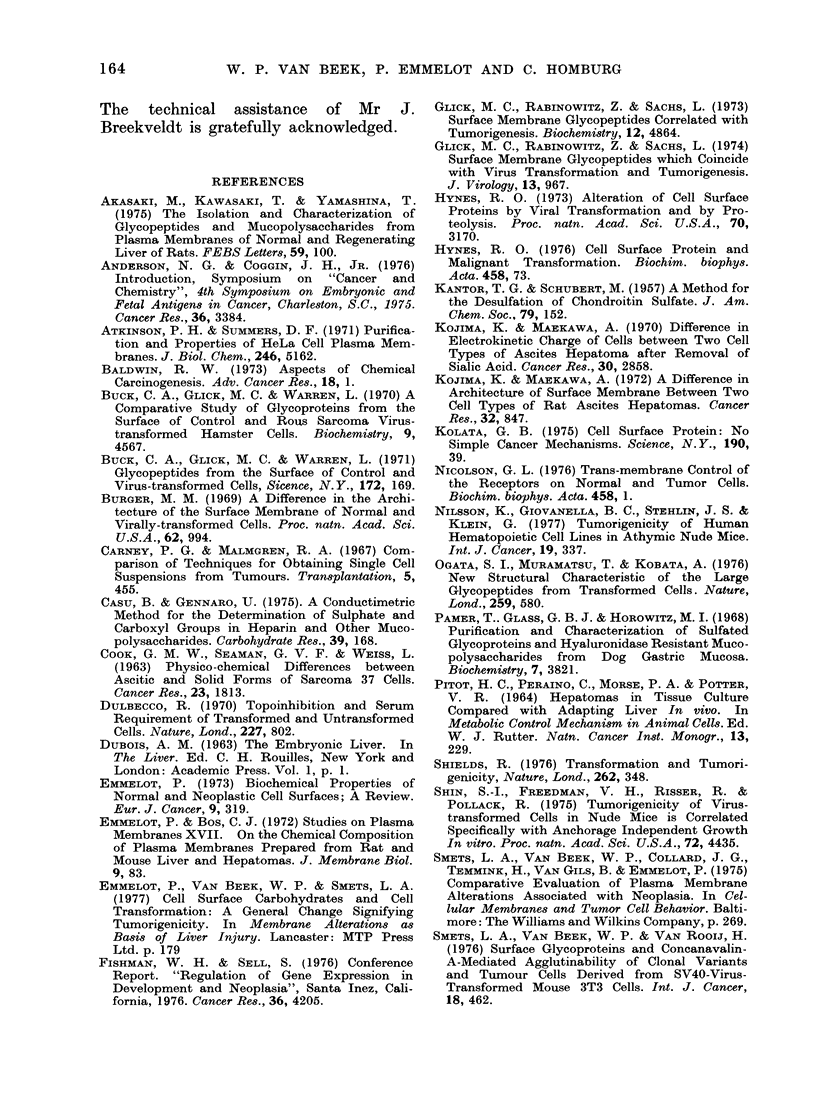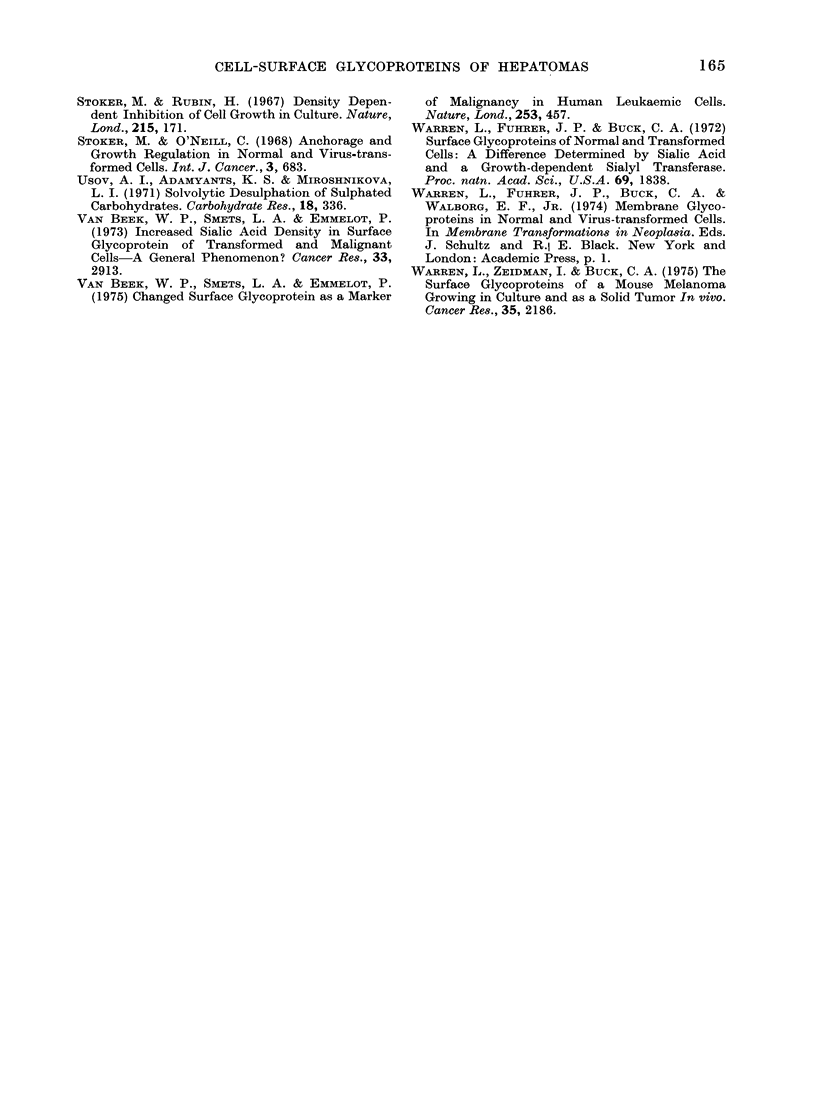# Comparison of cell-surface glycoproteins of rat hepatomas and embryonic rat liver.

**DOI:** 10.1038/bjc.1977.172

**Published:** 1977-08

**Authors:** W. P. van Beek, P. Emmelot, C. Homburg

## Abstract

Cell-surface glycoprotein of 3 rat hepatoma strains and late-embryonic liver was metabolically labelled in vivo with [3H]- or [14C]-fucose. Trypsinization of the cells and exhaustive pronase digestion of combined hepatoma-liver trypsinates followed by gel filtration over Sephadex-Biogel mixtures, yielded elution profiles that contained more early-eluting (high-mol.-wt.) glycopeptides for hepatomas than for liver. At least 3 factors were identified which acted to augment the fraction of early-eluting tumour glycopeptides: (a) increase of neuraminidase-sensitive sialic acid, (b) increase of neuraminidase-insensitive sialic acid that was sensitive to mild HCl hydrolysis, and (c) presence of sugar sulphate groups contributing to a restricted extent, relative to possible unknown factor(s). Whether (a), (b) or (c) operated depended on the hepatoma strain or its mode of growth. Notwithstanding these differences in the nature of the increase in early-eluting glycopeptides, the increase itself appears not to be due to growth per se, nor to an embryonic expression, but rather may serve as a marker of tumourigenicity.


*Br. J. Cancer* (1977) **36**, 157.

### COMPARISON OF CELL-SURFACE GLYCOPROTEINS OF RAT HEPATOMAS AND EMBRYONIC RAT LIVER

W. P. VAN BEEK, P. EMMELOT AND C. HOMBURG

*From the Division of Cell Biology, Antoni van Leeuwenhoek-Huis, The Netherlands Cancer Institute, Amsterdam, The Netherlands*

Received 1 March 1977 Accepted 6 April 1977

**Summary.—Cell-surface glycoprotein of 3 rat hepatoma strains and late-embryonic liver was metabolically labelled *in vivo* with [$^3$H]- or [$^{14}$C]-fucose. Trypsinization of the cells and exhaustive pronase digestion of combined hepatoma-liver trypsinates followed by gel filtration over Sephadex-Biogel mixtures, yielded elution profiles that contained more early-eluting (high-mol.-wt.) glycopeptides for hepatomas than for liver. At least 3 factors were identified which acted to augment the fraction of early-eluting tumour glycopeptides: (*a*) increase of neuraminidase-sensitive sialic acid, (*b*) increase of neuraminidase-insensitive sialic acid that was sensitive to mild HCl hydrolysis, and (*c*) presence of sugar sulphate groups contributing to a restricted extent, relative to possible unknown factor(s). Whether (*a*), (*b*) or (*c*) operated depended on the hepatoma strain or its mode of growth. Notwithstanding these differences in the nature of the increase in early-eluting glycopeptides, the increase itself appears not to be due to growth *per se*, nor to an embryonic expression, but rather may serve as a marker of tumourigenicity.**

THE cell surface seems to be intimately involved in the establishment of functional contact relations between cells with respect to recognition, adhesion and control of proliferation (Emmelot, 1973; Nicolson, 1976). Since these properties are impaired in neoplastic cells it may follow that surface changes are related to, and perhaps even be instrumental in neoplastic cell behaviour.

Initially, a number of phenomena such as loss of a high-mol.-wt. plasma-membrane protein (Hynes, 1973), increased concanavalin A agglutination (Burger, 1969), loss of density-dependent inhibition of movement and growth (Stoker and Rubin, 1967), decreased growth dependence on serum concentration (Dulbecco, 1970) and growth in semi-solid agarose (Stoker and O'Neill, 1968 seemed to distinguish between normal and transformed cells.

However, more extended studies have recently shown that these phenomena are not, or are not generally, characteristic of tumourigenic cells (Kolata, 1975; Shin *et al.*, 1975; Hynes, 1976; Nicolson, 1976; Shields, 1976; Smets, Van Beek and Van Rooij, 1976; Nilsson *et al.*, 1977). These findings underline the importance of extending investigations to several cell systems.

A more promising feature for distinguishing normal from malignant cells, in view of its more general occurrence, is the change recorded in membrane glycopeptides, following differential fucose labelling of normal and tumour cells (Buck, Glick and Warren, 1970, 1971; Glick, Rabinowitz and Sachs, 1973, 1974; Van Beek, Smets and Emmelot, 1973, 1975; Warren *et al.*, 1974; Smets *et al.*, 1975).

Since most of these experiments were carried out *in vitro* it was considered important to study the change in tumours grown and labelled *in vivo*. For *in vivo*

studies, suitable normal controls are a matter of concern, since the glycopeptides derived from cells of different organs of the rat exhibit differences in molecular distribution after gel filtration (unpublished observation). Since Warren, Zeidman and Buck (1975) lacked the corresponding *in vivo* normal cells, they compared melanomas grown and labelled *in vivo* with unrelated normal tissue such as liver and lung and tissue culture cells.

The present paper reports the results of the entirely *in vivo* comparison of three solid rat hepatomas with late-embryonic rat liver. The latter tissue is a vital control in view of the many findings that tumours, including hepatomas, reacquire embryonic characters, including cell-surface antigens (Baldwin, 1973; Anderson and Coggin, 1976; Fishman and Sell, 1976). The use of these tumours in the present experiments allowed the identification of at least 3 components that may increase the apparent molecular weight of tumour glycopeptides.

### MATERIALS AND METHODS

*Growth and labelling of cells and tumours.*—The Novikoff hepatoma strain $N_1S_1$–67 and Reuber H–35 hepatoma have been obtained as tissue-culture cell lines from Dr H. Van Rijn (State University of Utrecht) and maintained as described previously (Pitot *et al.*, 1964; Van Beek *et al.*, 1973). For the present experiments, $5 \times 10^6$ Novikoff hepatoma cells and $18 \times 10^6$ Reuber hepatoma cells were transferred to the peritoneal cavity of 3-months-old Sprague–Dawley (SD) and AXC, female rats, respectively, the former rats having received 450 rad whole-body irradiation. The Novikoff hepatoma yielded tumours that grew as minute "grapes", palpable within 1 week; this tumour is designated ST-1. The Reuber hepatoma also yielded grape-like tumours, palpable after about 3 weeks.

Another Novikoff hepatoma $N_1S_1$–67 strain was obtained through the courtesy of Dr E. F. Walborg Jr., The University of Texas, M.D. Anderson Hospital and Tumor Institute, at Houston. This tumour was routinely maintained in the ascites form by serial passages of 1 ml ascites fluid 8 days after transplantation. S.c. injection of 0·5 ml of ascites fluid resulted in a solid tumour nodule (reaching a diameter of 0·5–1·0 cm in about 2 weeks), referred to as ST-2.

L-[$^3$H]-fucose generally labelled (4·8 Ci/mmol; New England Nuclear, Boston, Mass.) was administered by i.p. injection, or directly into the solid tumours, as soon as tumour processes became palpable or had reached the above-mentioned diameter (3 tumour-bearing rats per experiment). The rats received 200 $\mu$Ci, followed after 24 h by another 100 $\mu$Ci. Pregnant SD females (3 months old) received on Day 18 of their pregnancy an i.p. injection of 100 $\mu$Ci L-[1-$^{14}$C]-fucose (60 mCi/mmol; The Radiochemical Centre, Amersham, England), followed after 24 h by another 50 $\mu$Ci. Twenty-four hours after the last injection, the rats were killed and the embryonic livers collected and washed with *N*-2-hydroxyethyl piperazine-N′-2-ethane-sulphonic acid-buffered Hanks' salt solution, pH 7·3 (HHS).

*Preparation and chromatography of glycopeptides.*—Solid hepatomas and liver tissue were dissociated by careful mincing with scissors in ice-cold HHS to small pieces of tissue of about 1–2 mm$^3$. The minced tissues were washed $\times 7$ by centrifugation at 800 *g* and resuspension in ice-cold HHS. Preparations from 5 embryonic livers and from hepatomas, both corresponding to 3–5 g wet-weight of tissue, were incubated with 90 ml HHS containing 0·25% trypsin (twice crystallized, type III; Sigma Chemical Co., St. Louis, Mo., U.S.A.) in Erlenmeyer flasks shaken for 60 min at 60 strokes per min at 35 °C.

This method was varied by a stepwise incubation of the tissue pieces with trypsin-containing HHS (90 ml total) the tissue pieces being treated successively with three 30-ml portions of digestion medium for 20 min, followed each time by separation of liberated cells and medium from the remaining tissue pieces, which were then incubated with 30 ml fresh digestion medium. The three 30-ml portions of medium were combined and processed further, the results being similar to those obtained with the above-mentioned continuous incubation. (Mincing of tissue and the second of the above-mentioned trypsin incubation procedures under essentially similar conditions have previously been used to obtain single cells from solid tumours (Garney and Malmgren, 1967).) In our hands, trypsinization of the hepatoma tissue pieces tended to damage the cells, but any intracellular membranes thus released would not influence the results, since these membranes of transformed cells exhibit the same type of glycopeptide change as does the surface membrane (Warren *et al.*, 1974, 1975). Moreover, fucose preferentially labels surface glycoprotein (Atkinson and Summers, 1971) and previous experiments with lymphoblasts (Van Beek *et al.*, 1973) have shown that "leaking" does not interfere with the results. The trypsinates thus obtained were filtered through cheesecloth, and further processed as described by Van Beek *et al.* (1973), including digestion by pronase (B grade, Calbiochem. A.G., Luzern, Switzerland) and neuraminidase (from *Vibrio cholerae;* Behringwerke A.G., Marburg/Lahn, Germany; the enzyme is multispecific towards the various O-glycosidically-linked sialyl bonds).

As an alternative to neuraminidase digestion, lyophilized pronase-digested glycopeptides were treated with 5 ml 0·01N HCl for 90 min at 80 °C. The reaction was terminated by cooling to room temperature and neutralization with NaOH, followed by dialysis (three-eighths-inch cellulose tubing; A. H. Thomas Co., Philadelphia, Pa., U.S.A.) at 6 °C for 24 h against twice-distilled water. The non-dialyzable material was lyophilized and stored at −40 °C prior to chromatography.

The freeze-dried glycopeptide preparation of the H-35 hepatoma, after mild HCl hydrolysis, was suspended in dry methanol containing 0·02M HCl (introduced by passing dry HCl gas through absolute methanol), for transesterification of any sulphate groups present in the glycopeptides (modified from Kantor and Schubert, 1957). To remove traces of water, 10 drops of 2,2-dimethoxypropane (I.C.N. Pharmaceuticals Inc., New York) were added to the suspension which was then shaken for 5 h at 60 °C. The reaction was stopped by neutralization with NaOH and cooling to room temperature. Methanol was removed under reduced pressure and the residue was solubilized in and dialyzed against twice-distilled water for 24 h at 6 °C, lyophilized, taken up in the usual eluent (Van Beek *et al.*, 1973), and stored at −40 °C until chromatography. In all cases, gel filtration was performed with 1-ml samples on a 2 : 1 mixture of Bio-Gel P10 (200–400 mesh; Bio-Rad Laboratories, Richmond, California) and Sephadex-G50 fine (Pharmacia, Uppsala, Sweden) as described (Van Beek *et al.*, 1973). Experiments were repeated 3 times with similar results.

In the elution profile, a vertical shift indicates a change in amount of material, and a horizontal one a change in mol. wt. or size. A profile is obtained by plotting the percentage radioactivity present in each fraction, with the radioactivity of the total eluate taken as 100. Thus the profiles illustrate relative amounts of fucose-labelled glycopeptides of various molecular weights and, since the columns were not overloaded in our experiments, a profile is—and was checked to be—independent of the amount of material brought on the column, and therefore suits comparative analysis. (For estimated mol. wts, see Ogata, Muramatsu and Kobata, 1976).

### RESULTS

Trypsinization of the hepatoma and liver cell preparations released 19–30% of the total amount of labelled fucose incorporated by the various tissues, in accord with previous findings on *in vitro* labelled cells (Van Beek *et al.*, 1973). Each of the trypsinates of Novikoff hepatoma (ST-1 and ST-2) and Reuber H-35 hepatoma cells, labelled *in vivo* with [$^3$H]-fucose, was combined with trypsinate of late-embryonic rat liver cells, also labelled *in vivo* but with [$^{14}$C]-fucose, and processed with pronase as described in the Materials and Methods section.

Gel filtration of the final glycopeptides obtained from each of these 3 pairs of cells yielded profiles in which the tumour-derived glycopeptides eluted ahead of the liver glycopeptides (Figs. 1A, 2A and 3A, Peak II).

The 3 hepatoma glycopeptide preparations were unequally sensitive to neuraminidase pretreatment in terms of their subsequent elution behaviour. In the case of the Novikoff ST-1 hepatoma, neuraminidase caused a shift in elution profile towards lower-mol.-wt (smaller sized) material (Fig. 1B, Peaks II and III moving into Peaks IV and V) that showed

FIG. 1.—Elution profiles of surface glycopeptides from the solid Novikoff rat hepatoma (ST-1) and late-embryonic rat liver. The hepatoma was labelled *in vivo* with [$^{3}$H]-fucose (●——●), the liver with [$^{14}$C]-fucose (○——○). A, pronase-digested glycopeptides; B, following neuraminidase treatment of A; C, following mild acid (HCl) hydrolysis of A. BD = blue dextran 2000.

the same elution profile as the neuraminidase-pretreated liver glycopeptides. This coincidence of elution profile indicated that the original difference in profiles resulted from an increased density of neuraminidase-sensitive sialyl groups in the tumour as compared with the liver glycopeptides.

However, no such coincidence of elution profiles of tumour and liver glycopeptides was obtained after neuraminidase pretreatment of the 2 other hepatomas, Novikoff ST-2 (Fig. 2B, Peak II moving into Peak III, increase of Peak IV and V) and Reuber H-35 (Fig. 3B, no effect on tumour glycopeptides). Although the liver control profiles in Figs. 1B and 2B might suggest an incomplete digestion by neuraminidase in the experiment of Fig. 2B, other similar experiments confirmed the difference in neuraminidase-insensitive sialic acid between ST-1 and ST-2 glycopeptides. Thus, sensitivity to neuraminidase, as judged by the shift to lower-mol.-wt. regions, decreased in the order Novikoff ST-1 > Novikoff ST-2 > Reuber H–35 hepatomas.

Previously, it has been noted (Emmelot and Bos, 1972) that about 30% of the sialic acid of plasma membranes isolated from solid rat hepatoma (and adult liver) was insensitive to neuraminidase. Accordingly, the presence of a neuraminidase-resistant sialic acid fraction might cause, or contribute to, the lack of effect of neuraminidase in the last 2 experiments. Therefore, a mild procedure for removing sialic acid by chemical hydrolysis, *i.e.* 90 min in 0·01N HCl at 80 °C, was devised. This procedure is considered to be specific for sialic acid for the following reasons: first, of the glycosyl bonds present in oligo-saccharide moieties, the sialyl bonds are the ones most sensitive to acid hydrolysis, followed by fucosyl bonds (Pamer, Glass and Horowitz 1968); and, secondly, at the most only 10% of the fucose label of the present preparations was lost by the mild HCl hydrolysis. Its application to the pronase-digested glycopeptides led to the following results:

(*a*) Novikoff ST-1 (Fig. 1C): The elution profiles of hepatoma and liver glycopeptides coincided. However, in both profiles Peak IV was similarly decreased and Peak V increased, relative to the results after neuraminidase pretreatment (Fig. 1B). These parallel shifts indicated the additional presence of some small and about equal amounts of

FIG. 2.—Elution profiles of surface glycopeptides from the solid Novikoff rat hepatoma (ST-2) and late-embryonic rat liver. For explanation see legend of Fig. 1.

neuraminidase-insensitive, but weak HCl-sensitive sialic acid in both the hepatoma and liver glycopeptides.

(*b*) Novikoff ST-2 (Fig. 2C): The mild HCl hydrolysis now led to the coincidence of tumour and liver elution profiles, which were similar in shape to those obtained in the previous experiment of Fig. 1C. This indicated that the original difference (Fig. 2A) between the Novikoff ST-2 and liver elution profiles mainly arose from an increased amount of neuraminidase-insensitive sialic acid in the tumour glycopeptides.

(*c*) Reuber H-35 (Fig. 3C): In this case the mild HCl hydrolysis had hardly any effect (this finding adds to the specificity of the hydrolytic method as argued above) as judged from the hepatoma elution profile, emphasizing the difference from the liver control. Hence in this case neither neuraminidase- nor weak acid-sensitive sialic acid (Figs. 3B and 3C, respectively) appeared to determine the difference between tumour and liver elution profiles.

In order to study whether sugar sulphates might be involved, a very mild transesterification reaction using dry methanol (containing 0·02M HCl), which should yield methylsulphate into solution, was carried out on the mild HCl-treated glycopeptides, as described in Materials and Methods. As shown in Fig. 3D, the restricted change obtained in the elution profile of the tumour, but not of the liver glycopeptide material, indicated that the presence of sulphate groups in the tumour glycopeptide was only to a limited extent responsible for the difference in elution profiles. Raising the acid concentration of the reaction mixture by using 0·04 or 0·06M HCl (the latter as used by Kantor *et al.*, 1957) resulted in a considerable loss of the fucose label. As the condition (0·02M HCl) might have been insufficient for complete transesterification, more specific conditions (Usov, Adamyants and Miroshnikova, 1971; Casu and Gennaro, 1975) are being studied.

### DISCUSSION

A change in a cell property that may differentiate normal from tumour cells—and thus may be used for detecting malignancy—should be studied in many cell systems, different with respect to cell type, mode of growth, species and oncogenic determinant, before its general occurrence can be accepted (Emmelot, 1973; Nicolson, 1976). This approach should be distinguished from the one that aims at establishing the functional significance of the phenomenon in molecular terms. The present paper contributes to both aspects.

First, the typical shift in the elution profile demonstrated here for 3 rat hepatomas relative to late-embryonic rat liver was obtained with materials that were differentially labelled *in vivo*. Accordingly, the glycopeptide alteration that is recorded represents a change in glycoprotein occurring *in vivo* that is not due to *in vitro* conditions such as have been used in most previous experiments, which might have influenced the results. It is very well documented that, in mammals, haemocytoblasts are invading all the hepatic parenchyma during embryonic life. This haemopoiesis attains its maximal activity towards 2/3 of the way through gestation, then regresses rapidly, resulting in a few disseminated islands of haemopoietic tissue at birth (Du Bois, 1963). Hardly any contamination of the latter tissue can be expected in our experiment using late embryonic liver as a control for hepatomas. Furthermore, regenerating rat liver has also been used as a control for rat hepatoma to the same purpose and with the same result (Akasaki, Kawasaki and Yamashina, 1975; Smets *et al.*, 1975). Thus neither proliferation *per se* (*c.f.* also Van Beek *et al.*, 1975) nor embryonic expression, is the cause of the altered surface glycoprotein of the tumour cells. Instead, this change rather appears to be intimately associated with the tumorigenic condition of cells.

Secondly, the experiments demonstrate the occurrence of at least three categories of biochemical change that may underly the increase in the higher-mol.-wt. tumour glycopeptides.

*a. Neuraminidase-sensitive sialic acid*

This acts as the molecular-weight determinant in most cases of neoplastic cells which (tend to) grow as single cells or in suspension, such as transformed cells *in vitro* (Warren, Fuhrer and Buck, 1972; Van Beek *et al.*, 1973), ascites tumour cells *in vivo* (the ascites form of Novikoff hepatoma ST-2): Smets *et al.*, 1975; mouse lymphosarcoma (MBVIA) and thymus-derived leukaemic (GRSL) cells: Emmelot, Van Beek and Smets, 1977) and many human leukaemias (Van Beek *et al.*, 1975). In all these cases, pretreatment with neuraminidase abolishes the difference in elution profiles between neoplastic and normal glycopeptides.

In the present study, this category is represented by the Novikoff hepatoma ST-1 (Fig. 1A–C) which grows in the form of minute "grapes" in the peritoneal cavity. In this tumour, as in the late-embryonic liver, intercellular connections are loose and the tissues are easily disaggregated.

*b. Neuraminidase-insensitive sialic acid sensitive to mild HCl hydrolysis*

This markedly contributed to the molecular-weight increment of the glycopeptides obtained from the solid Novikoff ST-2 hepatoma which was grown as a single s.c. tumour nodule (Fig. 2A–C). However, when grown *in vitro* (Van Beek *et al.*, 1973) or in ascites form *in vivo* (Smets *et al.*, 1975), the glycopeptides of this tumour become sensitive to neuraminidase. Hence it appears that a change in sialic acid disposition, affecting the sensitivity to neuraminidase, accompanies the solid-ascites interconversion (*c.f.* also Cook, Seaman and Weiss, 1963; Kojima and Maekawa, 1970, 1972). Mode of growth, rather than tumour type (as found in other cases, Emmelot *et al.*, 1977) here determines the sensitivity to neuraminidase. This phenomenon is not confined to neoplastic tissues but has also been observed for regenerating rat liver when compared with rat liver cells *in vitro* (Smets *et al.*, 1975; *c.f.* also Warren *et al.*, 1975). Nevertheless, the enrichment in early-eluting glycopeptides is observed for ST-2 cells irrespective of a resistance of the control material towards neuraminidase treatment.

*c. Sugar-sulphate ester groups, and unknown factors*

In the case of the solid Reuber H-35 hepatoma, the glycopeptides are refractory to the action of both neuraminidase and mild HCl treatment (Fig. 3A–C). These hepatoma cells, when cultured *in vitro* and compared with rat liver cells (Van Beek *et al.*, 1973), do behave similarly (not shown) to the solid tumour as described here. This particular behaviour thus seems to be tumour-strain-specific. A similar type of resistance to neuraminidase and mild acid hydrolysis has been encountered for human chronic myelocytic leukaemia (Van Beek *et al.*, 1975).

The limited effect of the transesterification pretreatment (Fig. 3D) seems, within the conditions used, to exclude a major contribution of sulphate groups to the difference in elution profiles. Hence the glycopeptides of this category may also contain an at present unknown molecular-weight determinant or determinants, or sialic acid residues with a very specific disposition which renders them insensitive to mild acid hydrolysis.

Finally, despite the fact that the nature of the change causing the increase in higher-mol.-wt. (early-eluting) glycopeptides may differ, the finding that the many tumours studied (summarized by Emmelot *et al.*, 1977) all show this increase is unique. To our knowledge it is the only biochemical parameter that at the present moment generally distinguishes cancerous from normal cells.

We wish to thank Dr L. A. Smets for his help in discussing this work with us.

FIG. 3.—Elution profile of surface glycopeptides from the solid Reuber H-35 rat hepatoma and late-embryonic rat liver. For explanation see the legend of Fig. 1; Fig. 3D illustrates the elution profile of the glycopeptides obtained by mild HCl hydrolysis followed by transesterification as described under Materials and Methods.

The technical assistance of Mr J. Breekveldt is gratefully acknowledged.

### REFERENCES

AKASAKI, M., KAWASAKI, T. & YAMASHINA, T. (1975) The Isolation and Characterization of Glycopeptides and Mucopolysaccharides from Plasma Membranes of Normal and Regenerating Liver of Rats. *FEBS Letters*, **59**, 100.

ANDERSON, N. G. & COGGIN, J. H., JR. (1976) Introduction, Symposium on "Cancer and Chemistry", *4th Symposium on Embryonic and Fetal Antigens in Cancer, Charleston, S.C., 1975. Cancer Res.*, **36**, 3384.

ATKINSON, P. H. & SUMMERS, D. F. (1971) Purification and Properties of HeLa Cell Plasma Membranes. *J. Biol. Chem.*, **246**, 5162.

BALDWIN, R. W. (1973) Aspects of Chemical Carcinogenesis. *Adv. Cancer Res.*, **18**, 1.

BUCK, C. A., GLICK, M. C. & WARREN, L. (1970) A Comparative Study of Glycoproteins from the Surface of Control and Rous Sarcoma Virus-transformed Hamster Cells. *Biochemistry*, **9**, 4567.

BUCK, C. A., GLICK, M. C. & WARREN, L. (1971) Glycopeptides from the Surface of Control and Virus-transformed Cells, *Sicence, N.Y.*, **172**, 169.

BURGER, M. M. (1969) A Difference in the Architecture of the Surface Membrane of Normal and Virally-transformed Cells. *Proc. natn. Acad. Sci. U.S.A.*, **62**, 994.

CARNEY, P. G. & MALMGREN, R. A. (1967) Comparison of Techniques for Obtaining Single Cell Suspensions from Tumours. *Transplantation*, **5**, 455.

CASU, B. & GENNARO, U. (1975). A Conductimetric Method for the Determination of Sulphate and Carboxyl Groups in Heparin and Other Mucopolysaccharides. *Carbohydrate Res.*, **39**, 168.

COOK, G. M. W., SEAMAN, G. V. F. & WEISS, L. (1963) Physico-chemical Differences between Ascitic and Solid Forms of Sarcoma 37 Cells. *Cancer Res.*, **23**, 1813.

DULBECCO, R. (1970) Topoinhibition and Serum Requirement of Transformed and Untransformed Cells. *Nature, Lond.*, **227**, 802.

DUBOIS, A. M. (1963) The Embryonic Liver. In *The Liver*. Ed. C. H. Rouilles, New York and London: Academic Press. Vol. 1, p. 1.

EMMELOT, P. (1973) Biochemical Properties of Normal and Neoplastic Cell Surfaces; A Review. *Eur. J. Cancer*, **9**, 319.

EMMELOT, P. & BOS, C. J. (1972) Studies on Plasma Membranes XVII. On the Chemical Composition of Plasma Membranes Prepared from Rat and Mouse Liver and Hepatomas. *J. Membrane Biol.* **9**, 83.

EMMELOT, P., VAN BEEK, W. P. & SMETS, L. A. (1977) Cell Surface Carbohydrates and Cell Transformation: A General Change Signifying Tumorigenicity. In *Membrane Alterations as Basis of Liver Injury*. Lancaster: MTP Press Ltd. p. 179

FISHMAN, W. H. & SELL, S. (1976) Conference Report. "Regulation of Gene Expression in Development and Neoplasia", Santa Inez, California, 1976. *Cancer Res.*, **36**, 4205.

GLICK, M. C., RABINOWITZ, Z. & SACHS, L. (1973) Surface Membrane Glycopeptides Correlated with Tumorigenesis. *Biochemistry*, **12**, 4864.

GLICK, M. C., RABINOWITZ, Z. & SACHS, L. (1974) Surface Membrane Glycopeptides which Coincide with Virus Transformation and Tumorigenesis. *J. Virology*, **13**, 967.

HYNES, R. O. (1973) Alteration of Cell Surface Proteins by Viral Transformation and by Proteolysis. *Proc. natn. Acad. Sci. U.S.A.*, **70**, 3170.

HYNES, R. O. (1976) Cell Surface Protein and Malignant Transformation. *Biochim. biophys. Acta.* **458**, 73.

KANTOR, T. G. & SCHUBERT, M. (1957) A Method for the Desulfation of Chondroitin Sulfate. *J. Am. Chem. Soc.*, **79**, 152.

KOJIMA, K. & MAEKAWA, A. (1970) Difference in Electrokinetic Charge of Cells between Two Cell Types of Ascites Hepatoma after Removal of Sialic Acid. *Cancer Res.*, **30**, 2858.

KOJIMA, K. & MAEKAWA, A. (1972) A Difference in Architecture of Surface Membrane Between Two Cell Types of Rat Ascites Hepatomas. *Cancer Res.*, **32**, 847.

KOLATA, G. B. (1975) Cell Surface Protein: No Simple Cancer Mechanisms. *Science, N.Y.*, **190**, 39.

NICOLSON, G. L. (1976) Trans-membrane Control of the Receptors on Normal and Tumor Cells. *Biochim. biophys. Acta.* **458**, 1.

NILSSON, K., GIOVANELLA, B. C., STEHLIN, J. S. & KLEIN, G. (1977) Tumorigenicity of Human Hematopoietic Cell Lines in Athymic Nude Mice. *Int. J. Cancer*, **19**, 337.

OGATA, S. I., MURAMATSU, T. & KOBATA, A. (1976) New Structural Characteristic of the Large Glycopeptides from Transformed Cells. *Nature, Lond.*, **259**, 580.

PAMER, T.. GLASS, G. B. J. & HOROWITZ, M. I. (1968) Purification and Characterization of Sulfated Glycoproteins and Hyaluronidase Resistant Mucopolysaccharides from Dog Gastric Mucosa. *Biochemistry*, **7**, 3821.

PITOT, H. C., PERAINO, C., MORSE, P. A. & POTTER, V. R. (1964) Hepatomas in Tissue Culture Compared with Adapting Liver *In vivo*. In *Metabolic Control Mechanism in Animal Cells*. Ed. W. J. Rutter. *Natn. Cancer Inst. Monogr.*, **13**, 229.

SHIELDS, R. (1976) Transformation and Tumorigenicity, *Nature, Lond.*, **262**, 348.

SHIN, S.-I., FREEDMAN, V. H., RISSER, R. & POLLACK, R. (1975) Tumorigenicity of Virus-transformed Cells in Nude Mice is Correlated Specifically with Anchorage Independent Growth *In vitro*. *Proc. natn. Acad. Sci. U.S.A.*, **72**, 4435.

SMETS, L. A., VAN BEEK, W. P., COLLARD, J. G., TEMMINK, H., VAN GILS, B. & EMMELOT, P. (1975) Comparative Evaluation of Plasma Membrane Alterations Associated with Neoplasia. In *Cellular Membranes and Tumor Cell Behavior*. Baltimore: The Williams and Wilkins Company, p. 269.

SMETS, L. A., VAN BEEK, W. P. & VAN ROOIJ, H. (1976) Surface Glycoproteins and Concanavalin-A-Mediated Agglutinability of Clonal Variants and Tumour Cells Derived from SV40-Virus-Transformed Mouse 3T3 Cells. *Int. J. Cancer*, **18**, 462.

STOKER, M. & RUBIN, H. (1967) Density Dependent Inhibition of Cell Growth in Culture. *Nature, Lond.*, **215**, 171.

STOKER, M. & O'NEILL, C. (1968) Anchorage and Growth Regulation in Normal and Virus-transformed Cells. *Int. J. Cancer.*, **3**, 683.

USOV, A. I., ADAMYANTS, K. S. & MIROSHNIKOVA, L. I. (1971) Solvolytic Desulphation of Sulphated Carbohydrates. *Carbohydrate Res.*, **18**, 336.

VAN BEEK, W. P., SMETS, L. A. & EMMELOT, P. (1973) Increased Sialic Acid Density in Surface Glycoprotein of Transformed and Malignant Cells—A General Phenomenon? *Cancer Res.*, **33**, 2913.

VAN BEEK, W. P., SMETS, L. A. & EMMELOT, P. (1975) Changed Surface Glycoprotein as a Marker of Malignancy in Human Leukaemic Cells. *Nature, Lond.*, **253**, 457.

WARREN, L., FUHRER, J. P. & BUCK, C. A. (1972) Surface Glycoproteins of Normal and Transformed Cells: A Difference Determined by Sialic Acid and a Growth-dependent Sialyl Transferase. *Proc. natn. Acad. Sci., U.S.A.* **69**, 1838.

WARREN, L., FUHRER, J. P., BUCK, C. A. & WALBORG, E. F., JR. (1974) Membrane Glycoproteins in Normal and Virus-transformed Cells. In *Membrane Transformations in Neoplasia*. Eds. J. Schultz and R. E. Black. New York and London: Academic Press, p. 1.

WARREN, L., ZEIDMAN, I. & BUCK, C. A. (1975) The Surface Glycoproteins of a Mouse Melanoma Growing in Culture and as a Solid Tumor *In vivo*. *Cancer Res.*, **35**, 2186.